# Cost‐effectiveness analysis of primary human papillomavirus testing in cervical cancer screening: Results from the HPV FOCAL Trial

**DOI:** 10.1002/cam4.3864

**Published:** 2021-04-02

**Authors:** Ian Cromwell, Laurie W. Smith, Kim van der Hoek, Lindsay Hedden, Andrew J. Coldman, Darrel Cook, Eduardo L. Franco, Mel Krajden, Ruth Martin, Marette H. Lee, Gavin Stuart, Dirk van Niekerk, Gina Ogilvie, Stuart Peacock

**Affiliations:** ^1^ Canadian Centre for Applied Research in Cancer Control BC Cancer Research Institute Vancouver BC Canada; ^2^ British Columbia Cancer Agency Cancer Control Research BC Cancer Research Institute Vancouver BC Canada; ^3^ Canadian Agency for Drugs and Technologies in Health Ottawa ON Canada; ^4^ Faculty of Health Sciences Simon Fraser University Burnaby BC Canada; ^5^ BC Academic Health Sciences Network Vancouver BC Canada; ^6^ British Columbia Centre for Disease Control Vancouver BC Canada; ^7^ Division of Cancer Epidemiology McGill University Montreal QC Canada; ^8^ British Columbia Cancer Agency Cervical Cancer Screening Program Vancouver BC Canada; ^9^ Vancouver General Hospital Gynecologic Oncology Vancouver BC Canada; ^10^ Faculty of Medicine The University of British Columbia Vancouver BC Canada; ^11^ Women’s Health Research Institute BC Women’s Hospital Vancouver BC Canada

**Keywords:** cancer prevention, gynecological oncology, HPV, screening, viral infection, women's cancer

## Abstract

The Human Papillomavirus FOr CervicAL cancer (HPV FOCAL) trial is a large randomized controlled trial comparing the efficacy of primary HPV testing to cytology among women in the population‐based Cervix Screening Program in British Columbia, Canada. We conducted a cost‐effectiveness analysis based on the HPV FOCAL trial to estimate the incremental cost per detected high‐grade cervical intraepithelial neoplasia of grade 2 or worse lesions (CIN2+). A total of 19,009 women aged 25 to 65 were randomized to one of two study groups. Women in the intervention group received primary HPV testing with reflex liquid‐based cytology (LBC) upon a positive finding with a screening interval of 48 months. Women in the control group received primary LBC testing, and those negative returned at 24 months for LBC and again at 48 months for exit screening. Both groups received HPV and LBC co‐testing at the 48‐month exit. Incremental costs during the course of the trial were comparable between the intervention and control groups. The intervention group had lower overall costs and detected a larger number of CIN2+ lesions, resulting in a lower mean cost per CIN2+ detected ($7551) than the control group ($8325), a difference of ‐$773 [all costs in 2018 USD]. Cost per detected lesion was sensitive to the costs of sample collection, HPV testing, and LBC testing. The HPV FOCAL Trial results suggest that primary HPV testing every 4 years produces similar outcomes to LBC‐based testing every 2 years for cervical cancer screening at a lower cost.

## Graphical abstract

The Human Papillomavirus FOr CervicAL cancer (HPV FOCAL) trial is a large randomized controlled trial comparing primary HPV testing to liquid‐based cytology for cervical cancer screening. We conducted a cost‐effectiveness analysis alongside the HPV FOCAL trial. Results showed the intervention group yielded a higher rate of detected cervical intraepithelial neoplasia of grade 2 or worse lesions (CIN2+) at a lower mean cost per CIN2+ detected ($7551) than the control group ($8325), a difference of −$773. Findings suggest that primary HPV testing is cost‐effective compared to liquid‐based cytology.

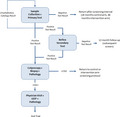

## 1 INTRODUCTION

Cancers of the cervix are relatively rare among Canadian women, representing less than 2% of incident cancers per year,^1^ with annual incidence decreasing an average of 1.8% over the past 20 years.^1^ The low and decreasing rate of cervical cancer owes a great deal to population‐level cervical cancer screening programs.^2^ Cervical cancer screening works by identifying and treating pre‐cancerous lesions, that is, cervical intraepithelial neoplasia (CIN)^3^ before they progress to cervical cancer. If a high‐grade CIN, grade 2 or greater (CIN2+) is detected, treatment is required. By detecting and removing pre‐cancerous lesions before they develop into invasive cancers, screening programs can reduce the incidence of cervical cancer.^2^ The Society of Obstetricians and Gynaecologists of Canada has recently urged for Canada to become the first country to eliminate cervical cancer through a coordinated prevention strategy that includes screening.^4^

The Human PapillomaVirus FOr CervicAL Cancer (HPV FOCAL) Trial (ISRCTN 79347302) is a large randomized controlled trial (RCT) conducted in British Columbia (BC), comparing the efficacy of primary HPV testing to liquid based cytology (LBC) for cervical cancer screening.^5^ The main trial objective was to compare the rates of CIN3+ 48 months after baseline screening with primary HPV versus LBC. Following the publication of the FOCAL Trial results^6^ and other emerging evidence of clinical effectiveness, the US Preventive Services Task Force has recommended the use of primary HPV testing instead of conventional Pap cytology.^7^

Early detection and treatment of high‐grade CIN has been shown to be an effective and cost‐effective method of reducing cervical cancer rates^8^, ^9^; however, screening strategies for early detection are evolving to reflect the well‐established role that human papillomavirus (HPV) plays in the development of virtually all cervical cancers.^10^, ^11^, ^12^, ^13^ HPV‐based screening has improved sensitivity over cytology testing and may also have implications for the cost‐effectiveness of cervical cancer screening programs.^8^, ^24^ Differences in test cost, rate of false and/or indeterminate results, time interval between routine screenings, and triage strategies may all produce changes in resource utilization and cost.

The HPV FOCAL trial was specifically designed to examine HPV testing as the primary screening modality for cervical cancer.^5^ Although cervical screening guidelines from a number of organizations have recommended primary HPV testing based on the natural history of cervical cancer, cross‐sectional studies, and modeling studies where HPV‐based screening was one element of a screening group, none of these studies were specifically designed to examine HPV testing as the primary screening modality.^5^ The HPV FOCAL trial is the first North American trial to compare cervical cancer screening modalities of primary HPV testing and LBC for the detection of precancerous lesions,^6^ and is the first trial in North America designed to allow the estimation the cost‐effectiveness of primary HPV testing compared to LBC using a within‐trial design.

The objective of this study here was to estimate the costs and effectiveness of using primary HPV testing, vs. primary testing using LBC in a population‐based cervix screening program, conducted alongside the HPV FOCAL trial.

## 2 METHODS

A full description of the FOCAL Trial population and methods is described elsewhere.^5^, ^6^, ^25^, ^26^ Briefly, eligible participants were women residing in BC, aged 25 to 65 who had not had a Pap smear in the last 12 months, were not pregnant at the baseline screen, were not HIV positive or receiving immunosuppressive therapy, and had no history of: CIN2+ in the past 5 years; invasive cervical cancer ever; or total hysterectomy. Women who met inclusion criteria and were patients of one of ~200 collaborating care providers in Metro Vancouver and Greater Victoria were invited to participate. Informed consent was obtained from all participants. Ethics approval was obtained from University of British Columbia Clinical Research Ethics Board (H06‐04032).

**FIGURE 1:**
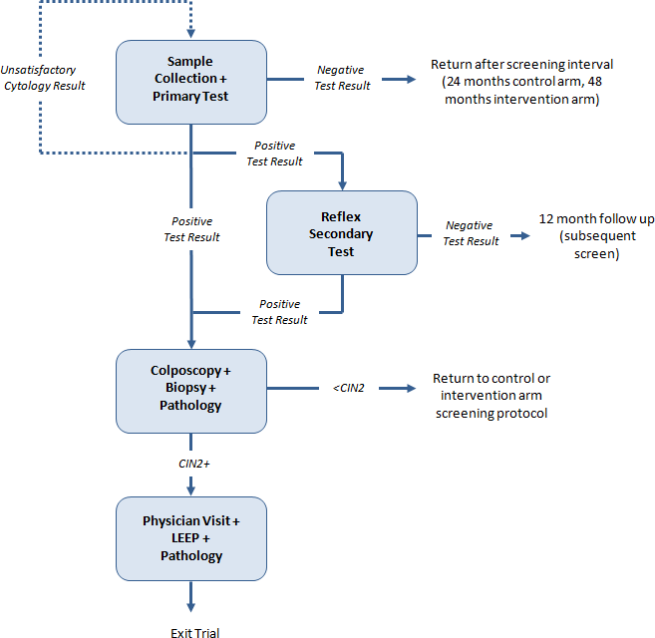
Resource utilization in the FOCAL Trial. The complete HPV FOCAL Trail Schematic is published in Ogilvie et al. (2017)^6^. Primary Test: HPV test in intervention group, LBC in control group. Secondary Test: LBC in intervention group, HPV in control group. CIN, Cervical Intraepithelial Neoplasia; LEEP, Loop Electrosurgical Excision Procedure

Participating women were randomized to one of three study groups—an intervention group, a control group, and a safety group. This analysis involves only the results of the intervention and control groups (the safety group was closed early once safety was demonstrated). Women randomized to the intervention group received baseline HPV testing. Baseline HPV‐negative women were recalled for exit at 48 months where they received HPV and cytology co‐testing. If baseline results were HPV positive, reflex LBC testing was done. If atypical squamous cells of undetermined significance (ASCUS) or greater, they were referred to colposcopy; if the LBC was negative, they were recommended to return in 12 months for repeat HPV and LBC testing. Women randomized to the control group received baseline LBC testing and if LBC negative were recalled at 24 months for screening with LBC and if negative at the 24‐month screen, were then recalled for exit screening at 48 months with HPV and LBC co‐testing. Those who were ASCUS at baseline received reflex HPV testing and if HPV positive, were then referred for colposcopy; and if cytology negative, were requested to return in 12 months for repeat LBC testing. Those with baseline LBC results low‐grade squamous intraepithelial lesions or greater (≥LSIL), or those with three consecutive LBC unsatisfactory results were referred for colposcopy and biopsy and managed based on the results. Women with CIN2+ were referred for management according to BC Cancer Cervix Screening Program provincial guidelines.^3^ Women in both the intervention and control groups who attended the 48‐month exit screen received both HPV and LBC co‐testing and were referred for colposcopy if they returned a positive result on either test. Those who were both HPV and cytology negative at exit screening were returned to routine screening through the provincial program (see Figure 1).

Resources utilized by participants in both study groups were collected throughout the course of the trial. At each screening appointment, participants underwent a pelvic examination and collection of a cervical specimen. The sample underwent LBC and/or HPV testing (depending on the study group, primary test result, or timepoint in the study). Women with positive samples could be re‐tested, and depending on study arm and result, women with positive HPV and/or LBC results were either recommended for colposcopy or to return for repeat testing in 12 months. If a CIN2+ lesion was detected at colposcopy, the woman was referred for standard of care management which typically includes excision therapy (loop electrosurgical excision procedure; LEEP) to remove the lesion. Not all women referred for colposcopy attended; accordingly, colposcopy resources were counted on an ‘intent to treat’ basis.^27^ Women who were lost to follow‐up were assumed to have not developed a lesion within the 48‐month follow‐up period. Women were followed by their colposcopist and or provider after CIN2+ diagnosis.^3^ Per trial protocol, women were discharged from the trial when a CIN2+ lesion was detected (Table 1).

**TABLE 1:** Resource utilization in the HPV FOCAL Trial

Appointment time	Participants screened	Samples collected	HPV tests	Cytology tests	Colposcopy referrals	CIN2+ detected
Intervention group						
Enrollment	9552	9655	9655	799	305	98
12 months	532	568	568	561	239	49
24 months	–	–	–	–	–	–
36 months	–	–	–	–	–	–
48 months	8296	8700	8700	8683	469	48
Total	9552	18923	18923	10043	1013	195
Control group						
Enrollment	9457	9860	89	9860	279	89
12 months	53	63	0	63	11	1
24 months	8040	8333	67	8333	145	38
36 months	35	35	0	35	2	0
48 months	8139	8445	8445	8445	513	62
Total	9457	26727	8601	26727	913	190

Note

Results are presented for full trial, including 48‐month exit co‐testing.

Unit costs were estimated for each resource used in the trial. The cost of a screening appointment was directly derived from reimbursement rates from the BC Medical Services Plan (MSP), which is the provincial insurance provider for medically necessary services in BC.^28^ The costs of LBC and HPV testing were estimated from FOCAL Trial and BC Cervix Screening Program costs. The costs of colposcopy were estimated as the cost of a gynecological consultation appointment,^28^ plus the per‐minute salary of a four year Level 2 Licensed Practical Nurse multiplied by an estimated 15‐minute procedure length.^29^ Cost of cervical biopsy was directly derived from MSP rates.^28^ Excision therapy (LEEP) was also directly derived from MSP rates plus a pathology‐related technical fee.^28^, ^30^ The cost of histology review by a pathologist for a diagnostic sample was estimated based on the assumption of a Level 4 equivalent (L4E) unit for each sample, with physician salary costs derived from the BC Provincial Service Contract agreement.^31^ All costs and their sources are described in Table 2.

**TABLE 2:** Screening resource unit costs

Parameter	Mean	Source
Cost of HPV test	$30.59	HPV FOCAL internal costing
Cost of sample collection	$24.28	MSP 14560^29^
Cost of liquid based cytology	$15.00	HPV FOCAL internal costing
Cost of colposcopy	$113.20	MSP 04012^29^ + 15 min LPN salary^30^
Cost of a physician visit	$59.06	MSP 04012^29^
Cost of excision therapy (LEEP)	$620.64	MSP 04620^29^
Cost of cervical biopsy	$52.20	MSP 04510^29^
Cost of sample pathology	$178.43	Procedural fee pathologist^31^, ^32^

Note

All $ in 2018 USD; 1 USD = 1.2957 CAD.

All costs are undiscounted.

Abbreviations: CIN, Cervical Intraepithelial Neoplasia; LEEP, Loop Electrosurgical Excision Procedure; MSP, British Columbia Medical Services Plan.

Total costs were estimated for each group, as well as the cost per detected lesion in each arm and the cost per trial participant in each group. It is important to note, the primary end‐point for this study was pre‐determined by the design of the HPV FOCAL Trial, as we are reporting within trial results here. The FOCAL Trial included exit co‐testing in the design of both arms and in its reporting of primary end‐points, which means our primary end‐points also include exit co‐testing.

That said, since the exit co‐testing (i.e., both LBC and HPV) at the 48‐month appointment is not representative of how screening would be conducted outside the trial, a secondary analysis was performed that excluded the results of 48‐month exit co‐testing. In this scenario, 48‐month results included only CIN2+ that were detected via the primary screening method (i.e., HPV testing in the intervention group, LBC in the control group) in the denominator. Resource use and costs attributable to the secondary screening method were excluded from the numerator. Costs attributable to a reflex secondary screening (due to positive primary) were included, while co‐testing costs that would not otherwise have occurred (due to negative primary) were excluded.

Ninety‐five per cent confidence intervals (CIs) around detected CIN2+ lesion counts were estimated from a Poisson distribution. Univariate threshold analyses were conducted to estimate the change in unit costs that would be necessary for the cost per lesion detected to be equivalent between the two screening strategies. All costs are expressed in 2018 US Dollars (USD), converted from Canadian Dollars using the annual exchange rate for 2018.^32^

## 3 RESULTS

Within‐trial resource use from the FOCAL Trial is presented in Table 2. One hundred ninety‐five (195) CIN2+ lesions were detected in the intervention group (95% CI: 169–224). One hundred ninety (190) CIN2+ lesions were detected in the control group (95% CI: 164–219). The number of CIN2 lesions (106 vs. 97) and CIN3 lesions (89 vs. 93) was comparable between the intervention and control groups, respectively.

Total cost within the intervention group was estimated at $1,472,445 or $154 per enrolled participant. Total cost within the control group was estimated at $1,581,750 or $167 per enrolled participant. The mean cost per detected CIN2+ lesion was $7551 versus $8325 for the intervention and control groups, respectively—a difference of ‐$773. The intervention protocol was less costly and more effective than the control protocol.

When analyzing trial results without co‐testing, the two arms had similar total costs ($1,346,368 in the intervention group, $1,332,870 in the control group), with a higher number of CIN2+ lesions detected in the intervention group (193) than in the control group (165). Cost per detected CIN2+ lesion was $6976 in the intervention group, and $8078 in the control group. The proportion of total cost attributable to co‐testing was lower in the intervention group, representing 8% of total costs vs. 15% of total costs in the control group (21% and 53% of 48‐month costs, respectively). Results are presented in Table 3, along with results excluding 48‐month co‐testing.

**TABLE 3:** HPV FOCAL Trial cost‐effectiveness results ($ in 2018 USD)

	Total costs	CIN2+ detected		Cost per CIN2+ detected		Cost per enrolled participant	
		Number	95% CI	Mean	95% CI	Mean	95% CI
Within trial							
Intervention group	$1,472,445	195	169–224	$7551	$6607‐$8758	$154	$135–$179
Control group	$1,581,750	190	164–219	$8325	$7223‐$9645	$167	$145–$194
Incremental	‐$109,305	5		‐$773		$−12	
No Co‐testing @ 48 months							
Intervention group	$1,346,368	193	166–220	$6976	$6361‐$8431	$141	$128–$170
Control group	$1,332,870	165	140–190	$8078	$7279‐$9879	$142	$126–$172
Incremental	‐$13,498	28		‐$1102		$−1	

Note

All $ in 2018 USD; 1 USD = 1.2957 CAD.

All costs and outcomes are undiscounted.

Baseline enrolment: Intervention group = 9552; Control group = 9457.

Univariate analysis suggests that the cost per detected CIN2+ results are sensitive to the cost of sample collection, HPV testing, and LBC testing, due in large part to the nearly‐equivalent costs and number of lesions found between the two groups over 48 months. Full results for univariate threshold analyses across the three different analytic perspectives are found in Table 4.

**TABLE 4:** Threshold analysis for unit cost values^a^

		Within Trial	No 48‐month Co‐testing
Value	Baseline unit cost		
Cost of HPV test	$30.59	$45.72	$42.40
Cost of collecting sample	$24.28	$6.63	$7.09
Cost of liquid based cytology	$15.00	$6.10	$7.61
Cost of colposcopy	$113.20	$5417.40	NT
Cost of physician visit	$59.06	>$100,000	NT
Cost of excision therapy (LEEP)	$620.64	>$100,000	NT
Cost of cervical biopsy	$52.20	$5356.40	NT
Cost of pathology review	$178.43	>$100,000	NT

Note

All costs are undiscounted.

All $ in 2018 USD; 1 USD = 1.2957 CAD.

Abbreviations: NT, No threshold value exists (i.e., cost‐effectiveness is robust to changes in this parameter).

^a^ If the unit cost exceeds the threshold value, the Intervention Group protocol in no longer cost‐saving compared to the Control Group protocol.

## 4 DISCUSSION

Our analysis suggests that despite the higher test cost, HPV‐based screening every four years is associated with lower costs and superior rates of CIN2+ detection compared to liquid‐based cytology screening every 2 years among women who regularly attend screening. This improvement in relative cost‐effectiveness was due primarily to the longer interval between tests, but also in part to a reduced need for re‐screening due to unsatisfactory samples, and a lower false positive rate at each screening appointment. The 48‐month exit co‐testing costs played a larger role in the control group, due to higher rates of CIN2+ detection from the HPV co‐test that was not detected by LBC. Results were sensitive to the costs of sample collection and testing. This suggests that changes in the cost and/or specificity of HPV testing could have an impact on the cost‐effectiveness of a system‐wide screening program.

The costs of LBC and HPV testing were estimated from internal FOCAL Trial costs. It is possible that the cost of HPV testing may come down if it is introduced on a population level, meaning HPV based‐screening may result in further cost savings to the public. Data on the costs of annual cytology follow‐up or periodic co‐testing following treatment for cases of CIN2+ (including LEEP) were not collected within the trial. As more cases of CIN2+ were detected and treated in the intervention arm, this may result in a slight over‐estimation of cost savings.

The consensus of Canadian^19^, ^33^, ^34^, ^35^ and international evidence^9^, ^36^ is that primary HPV testing is acceptably cost‐effective when compared to conventional cytology. Our trial endpoint was cost per CIN2+ lesion detected rather than incremental quality‐adjusted life‐years gained. As a result, our findings are not directly comparable to those findings. Our results do suggest, however, that screening led by HPV testing does not cost more than conventional screening, and is expected to be cost saving when implemented as regular practice.

Our results were based on a large population of screening participants within a centralized screening program. Nevertheless, there are some limitations to our findings. First, our screening population was observed in the context of a clinical trial, which included reminder calls and follow‐up that exceeds what would be current standard of care at the population level. This may slightly increase the costs and number of CIN2+ cases detected in both arms, but we do not anticipate it would affect our incremental cost‐effectiveness findings. Second, our comparator (LBC‐based screening every two years with reflex HPV testing for ASCUS results) is based on the FOCAL Trial protocol, not current standard of care in BC. At the time the FOCAL trial was developed and conducted, cytology testing every two years was the standard of care. This may slightly increase the number of CIN2+ cases detected in the control arm, and suggests our results may provide a conservative estimate of the incremental benefits of HPV testing when compared to standard care. Third, this evaluation was based on a two‐year screening interval for LBC cytology and a 4‐year interval for HPV testing. The US Preventive Services Task Force recently released a recommendation, based in part on FOCAL Trial results, that primary HPV testing every five years was preferable to conventional screening every 3 years.^7^ As a result, our findings cannot be directly compared with current US screening recommendations.

It is important to note that the number and timing of detected CIN2+ lesions differ between the trial groups. A larger number of lesions were detected in the HPV group in round one (baseline up to 12 month follow‐up), with a smaller number at 48‐month exit; this was reversed for the control group, with more CIN2+ detected in the control group at exit due to the HPV co‐test. This suggests our results may not fully reflect the impact of regular HPV testing in the long term (i.e., over the lifetime of a screening participant, with multiple consecutive screens).

Our results are also limited to the number of CIN2+ lesions detected within the FOCAL Trial because this was the end‐point for the RCT itself, and assume that women who are lost to follow‐up did not go on to develop a lesion. Consequently, our analysis may underestimate the total number of lesions detected. Given the similar event and dropout rates between the two arms, it is unlikely that this underestimation meaningfully affects the overall findings presented in this analysis.

Analysis of the HPV FOCAL Trial results suggests that a population‐level, cervical cancer screening using primary HPV testing with reflex cytology is equivalent or superior to primary LBC testing with reflex HPV testing, at detecting CIN2+ lesions among women who are regular participants in cervical cancer screening. Future investigations, using the HPV FOCAL Trial results, into the cost‐effectiveness of screening according to the USPTF guideline recommendation are also planned, as is the cost‐effectiveness of using a higher threshold (LSIL, HSIL) for colposcopy referral, and modeling incremental quality‐adjusted survival beyond the time horizon of the trial as the outcome of interest.

## ROLE OF THE FUNDER/SPONSOR

As part of its review and approval of the funding application, CIHR approved the design, analysis, and conduct of the study. The funder had no role in the collection, management, and interpretation of the data; preparation, review, or approval of the manuscript; and decision to submit the manuscript for publication.

## Data Availability

Author elects to not share data. Data are not shared because research is still underway.
